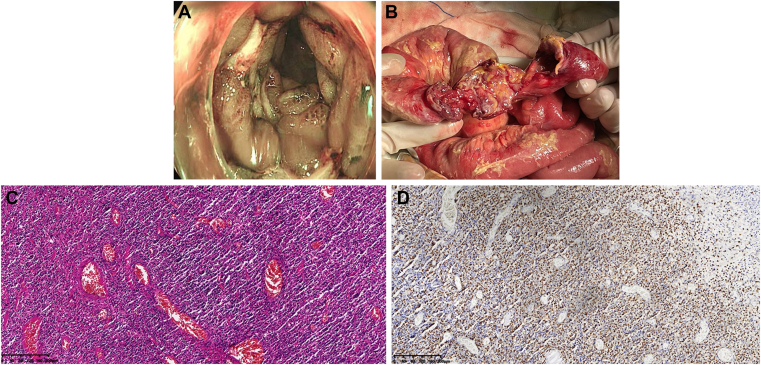# Extranodal Natural Killer/T-Cell Lymphoma Presenting as Intestinal Stenosis and Perforation

**DOI:** 10.1016/j.gastha.2026.100986

**Published:** 2026-04-30

**Authors:** Hongyang Li, Linbo Yao, Meiduo Ouzhu

**Affiliations:** 1 Tibet University School of Medicine, Lhasa, Tibet Autonomous Region, China; 2 Department of Gastroenterology, Tibet Autonomous Region People's Hospital, Lhasa, Tibet Autonomous Region, China

## Case Presentation

A 45-year-old man presented with 1 year of abdominal pain and intermittent hematochezia, with a 15-pound weight loss over 2 months. Examination revealed subxiphoid tenderness. Laboratory tests showed anemia (hemoglobin 102 g/L) and hypoalbuminemia (25.2 g/L). Colonoscopy demonstrated multiple segmental, circumferential nodular and polypoid mucosal elevations with ulceration and white exudate in the ascending and transverse colon, causing luminal stenosis (Figure A). After colonoscopic examination, he developed worsening abdominal pain and fever, with diffuse tenderness and rebound tenderness. Contrast-enhanced computed tomography showed thickened colonic walls at the hepatic and splenic flexures, free intraperitoneal air, and pelvic encapsulated effusion, suggesting perforation. Emergency surgery revealed an ileal perforation, and almost 10-cm segment of the ileum was thickened with ulcer and hyperemia (Figure B). Partial ileal resection was performed. Histopathology showed acute and chronic inflammation with ulceration and perforation, marked lymphoid hyperplasia, and angiocentric growth (Figure C). In situ hybridization for Epstein–Barr virus–encoded RNA was diffusely positive (Figure D). Immunohistochemical findings supported a diagnosis of extranodal natural killer/T-cell lymphoma. The patient was transferred to the hematology department for further treatment.

**Figure:**